# Supplementary material for: Integrative characterization of the near‐minimal bacterium Mesoplasma florum
Source: Mol Syst Biol. 2020 Dec 17;16(12):e9844. doi: 10.15252/msb.20209844 (PMC7745072; doi:10.15252/msb.20209844)
Supplement: Supplementary file 2 — Appendix [file MSB-16-e9844-s001.pdf]

Appendix

Integrative characterization of the near-minimal bacterium *Mesoplasma florum*

Dominick Matteau[1], Jean-Christophe Lachance[1], Frédéric Grenier[1], Samuel Gauthier[1], James M. Daubenspeck[2], Kevin Dybvig[2], Daniel Garneau[1], Thomas F. Knight[3], Pierre-Étienne Jacques[1], & Sébastien Rodrigue[1#].

[1]Département de biologie, Université de Sherbrooke, Sherbrooke, Québec, Canada.
[2]Department of Genetics, University of Alabama at Birmingham, Birmingham, Alabama, USA.
[3]Ginkgo Bioworks, Boston, Massachusetts, USA.

#Corresponding author. E-mail: sebastien.rodrigue@usherbrooke.ca

Running title: *Mesoplasma florum* characterization

This Appendix includes:

Supplementary Materials and Methods

Supplementary Text

Supplementary Figures S1-S10

Supplementary Table S1

Supplementary References

Supplementary Materials and Methods

Dry mass quantification

Dry mass quantification of *M. florum* was performed in quadruplicate and repeated three times using 20 ml exponential-phase cultures. Briefly, cultures were centrifuged at 10°C for 15 min at 7,900 x *g*, washed twice with cold PBS1X, and then transferred into microtubes pre-weighted using a Sartorius ME235P analytical scale. Microtubes containing cells were centrifuged at 10°C for 2 min at 21,100 x *g* and cell pellets were resuspended in PBS1X. Resuspended cells were then serially diluted in triplicate with PBS1X in a 96-well microplate and cell concentration was measured by flow cytometry (FCM) as described in the Growth kinetics assays section of Materials and Methods. Undiluted cell suspensions were then centrifuged at 10°C for 2 min at 21,100 x *g*, supernatants were removed, and cell pellets were dried at 80°C for ~36 hrs. Dried cell pellets were then weighted using a Sartorius ME235P analytical scale. The *M. florum* dry mass per cell was determined by dividing the mass of the dried cell pellet by the total number of cells present in the sample measured by FCM.

Protein mass quantification

Protein mass quantification of *M. florum* was performed in quadruplicate by fluorescence-based protein quantification of whole-cell lysates. Briefly, whole-cell lysates were prepared by centrifuging exponential-phase *M. florum* cultures at 10°C for 15 min at 7,900 x *g*. Cells were washed twice with cold PBS1X, and then resuspended in PBS2X. Colony forming units (CFUs) were measured in triplicate by spotting serial dilutions of the samples on ATCC 1161 solid medium and counting colonies after an incubation of 24-48 hrs at 34°C. Sodium deoxycholate was then added to the cell suspensions to obtain a final concentration of 0.4% (w/v) in PBS1X, and

cells were lysed using a Bioruptor UCD-200 sonication system (Diagenode) set at high intensity and 4°C for 35 cycles (30 sec on, 30 sec off). Protein concentration was measured using the CBQCA Protein Quantitation Kit (Molecular Probes, C-6667) according to the manufacturer's specifications. Fluorescence was measured using a Synergy HT microplate reader (BioTek) with the 485/20 and 528/20 nm excitation and emission filters, respectively. The total mass of protein per cell was determined by dividing the protein concentration of the sample by the cell concentration measured by CFU counts.

DNA mass quantification

DNA mass quantification of *M. florum* was performed in quadruplicate by fluorescence-based nucleic acid quantification of purified genomic DNA (gDNA). gDNA was extracted from exponential-phase *M. florum* cultures using the Zymo Quick-DNA MiniPrep Kit (Zymo Research, D3025) according to the manufacturer's specifications, with the exception that cells were sonicated in genomic lysis buffer using a Bioruptor UCD-200 sonication system (Diagenode) set at medium intensity and 4°C for 5 cycles (30 sec on, 30 sec off) prior to the column purification step. A purification control consisting of previously purified *M. florum* gDNA of known concentration (measured using Quant-iT PicoGreen dsDNA Assay Kit (Thermo Fisher Scientific, P7589)) was also performed in quadruplicate to evaluate purification efficiency. The DNA concentration of purified gDNA samples and controls was then measured by fluorescence-based quantification using the Quant-iT PicoGreen dsDNA Assay Kit (Thermo Fisher Scientific, P7589). Fluorescence was measured using a Synergy HT microplate reader (BioTek) with the 485/20 and 528/20 nm excitation and emission filters, respectively. The total mass of DNA per cell was determined by first normalizing the concentration of the purified *M. florum* gDNA by the purification efficiency, and then by dividing the normalized DNA concentration by the initial culture cell concentration

measured in triplicate by spotting serial dilutions on ATCC 1161 solid medium and counting colonies after an incubation of 24-48 hrs at 34°C.

RNA mass quantification

RNA mass per *M. florum* cell was quantified in quadruplicate as described in the Appendix DNA mass quantification section (see above), with the exception that cells were sonicated in QIAzol (QIAGEN) reagent, RNA was purified and treated with DNase I using the Direct-zol RNA MiniPrep Kit (Zymo Research, R2052), and RNA was quantified using Quant-iT RiboGreen RNA Assay Kit (Thermo Fisher Scientific, R11490) according to the manufacturer's specifications.

Carbohydrate mass quantification and monosaccharide composition analysis

The monosaccharide composition and mass quantification of *M. florum* carbohydrates was determined in quadruplicate by gas chromatography-mass spectrometry (GC-MS) performed on whole-cell lysates. Briefly, exponential-phase *M. florum* cultures were centrifuged at 10°C for 2 min at 21,100 x *g*, and then washed twice with cold PBS1X. Cells were centrifuged again, resuspended in molecular grade water, and CFUs were evaluated in triplicate by spotting serial dilutions on ATCC 1161 solid medium and counting colonies after a 24-48 hrs incubation at 34°C (in triplicate). Resuspended cells were then lysed using a Bioruptor UCD-200 sonication system (Diagenode) set at high intensity and 4°C for 35 cycles (30 sec on, 30 sec off). Whole-cell lysates were then dried by vacuum centrifugation, resuspended in 400 µl of 1.45 N methanolic HCl, and treated at 80°C overnight to generate the methyl glycosides. The methanolic HCl was removed by vacuum centrifugation, and samples were resuspended in 200 µl of methanol, followed by the addition of 25 µl of acetic anhydride and 25 µl of pyridine. The mixture was allowed to react for 30 min at room temperature and then evaporated under vacuum centrifugation. Samples were

sealed under argon and then trimethylsilylated using 50 µl of Tri-Sil (Fisher). Samples were finally analyzed using a Varian GC-MS in the electron ionization mode. The monosaccharide composition and concentration were determined by comparison with known standards ran as a standard curve (Sigma-Aldrich), and normalized using the protein concentration of the analyzed samples. Protein concentration was calculated by multiplying the number of CFUs present in the cell resuspension before the lysis step by the total protein mass per cell evaluated previously (see Appendix Protein mass quantification section).

Lipid mass quantification

Lipid mass quantification of *M. florum* was performed in quadruplicate by fluorescence-based phospholipid quantification of whole-cell lysates. Whole-cell lysates were prepared as described in the Appendix Protein mass quantification section (see above). The phospholipid concentration of whole-cell lysates (molarity) was measured based on choline quantification using the Phospholipid Assay Kit (Sigma-Aldrich, MAK122) according to the manufacturer's specifications. Fluorescence was measured using a Synergy HT microplate reader (BioTek) with the 530/25 and 590/35 nm excitation and emission filters, respectively. The number of moles of choline-positive lipids per *M. florum* cell was calculated by dividing the measured concentration of whole-cell extracts by the cell concentration evaluated by CFU counts. The total mass of lipids per cell was then inferred based on the lipidomic profile of *M. florum* (see Dataset EV8 and Lipid mass spectrometry section). Briefly, identified lipid species were categorized as either choline-positive or choline-negative species (Fahy *et al*, 2009), and the average molecular weight of each category was calculated from the relative abundance and theoretical molecular weight of each included species. The number of moles of choline-negative lipids was then calculated according to the abundance fraction of each category (~47% and ~53%, respectively), and the total mass per

cell of choline-positive and choline-negative lipids was calculated by multiplying the number of moles of each category by their respective average molecular weight. The total lipid mass per *M. florum* cell was finally obtained by adding up the mass per cell of both lipid categories.

Lipid mass spectrometry

The lipid composition of *M. florum* was determined by direct infusion-tandem mass spectrometry (DI-MS/MS). Sample preparation and analysis was executed by PhenoSwitch Bioscience (Sherbrooke, Canada). Briefly, an exponential-phase *M. florum* culture was centrifuged at 10°C for 2 min at 21,100 x *g* and washed three times with cold electroporation buffer (272 mM sucrose, 1 mM HEPES [pH 7.4]). Cells were centrifuged again, the supernatant was discarded, and lipids were extracted from the cell pellet by liquid-liquid extraction. Cells were resuspended in 640 µl of ethanol, vortexed for 10 min, and 320 µl of chloroform was added (ethanol/chloroform 2:1 [v/v]). The mixture was vortexed again for 10 min and the insoluble material was removed by centrifugation. The supernatant was transferred into a new microtube, 400 µl of water was added, and the mixture was vortexed for 10 min. Phases were separated by centrifugation and the bottom phase was transferred into a new microtube and washed with 500 µl of chloroform/methanol/water 3:48:47 (v/v/v). The washed bottom phase was then dried and reconstituted in a 1:1 dichloromethane/methanol solution containing 2 mM ammonium acetate, diluted 10 fold, and analyzed on a TripleTOF 5600 mass spectrometer (SCIEX) by direct sample infusion (25 µl) in the mobile phase (1:1 dichloromethane/methanol, 2 mM ammonium acetate). Lipids were analyzed in positive and negative modes using a MS/MS all method (1 m/z windows). Lipid species were identified using LipidView version 1.2 (SCIEX). Only species belonging to the confirmed and common lipid group with an abundance of at least 5% relative to the most abundant

identified species were considered significant and used in the determination of the total lipid mass per cell (see Dataset EV8).

Description of cell mass equations

Given a spherical *M. florum* cell with a certain diameter (d), its cell mass (CM) can be described as the product of its volume (V) and its buoyant density (D):

$$CM = V \times D \qquad (A.1)$$

Since the volume of a sphere (V) with a certain diameter (d) is given by the following equation:

$$V = \frac{\pi d^3}{6} \qquad (A.2)$$

The cell mass (CM) of *M. florum* can thus be described as follows:

$$CM = \frac{\pi d^3}{6} \times D \qquad (A.3)$$

Alternatively, the mass of a cell (CM) can also be expressed as the ratio of its dry mass (DM) and its dry mass fraction (DF), the latter given by subtracting the water mass fraction (WF) of a cell from its total mass fraction, i.e. 1:

$$CM = \frac{DM}{DF} \qquad (A.4)$$

or

$$CM = \frac{DM}{1 - WF} \qquad (A.5)$$

If we separate the dry mass (DM) of a spherical cell from its water content, then the cell mass (CM) can be written as the cell volume (V) minus the volume occupied by its dry mass (V_{DM}), to

which we multiply the density of water (approximated to 1.00 g/ml) and finally add the said dry mass (DM):

$$CM = (V - V_{DM}) \times 1 + DM \qquad (A.6)$$

Since the dry mass volume (V_{DM}) can be particularly difficult to measure, this variable can be substituted by the ratio of the dry mass (DM) and its specific density (D_{DM}), which gives the following equation:

$$CM = \left(V - \frac{DM}{D_{DM}}\right) \times 1 + DM \qquad (A.7)$$

Or, if we develop the cell volume (V) as given by equation A.2:

$$CM = \left(\frac{\pi d^3}{6} - \frac{DM}{D_{DM}}\right) \times 1 + DM \qquad (A.8)$$

Conversely, if we replace the cell dry mass (DM) in equation A.4 by the product of its volume (V_{DM}) and its specific density (D_{DM}), we obtain:

$$CM = \frac{D_{DM} \times V_{DM}}{DF} \qquad (A.9)$$

From this formula, the dry mass volume (V_{DM}) can be isolated and substituted in equation A.6:

$$V_{DM} = \frac{CM \times DF}{D_{DM}} \qquad (A.10)$$

and

$$CM = \left(V - \frac{CM \times DF}{D_{DM}}\right) \times 1 + DM \qquad (A.11)$$

Finally, we can substitute one of the cell mass (CM) of equation A.11 by the cell mass expression of equation A.3 and develop the cell volume (V) as in equation A.2, which generates a formula unifying the *M. florum* cell diameter (d), buoyant density (D), dry mass fraction (DF), total dry mass (DM), and dry mass specific density (D_{DM}):

$$CM = \left(\frac{\pi d^3}{6} - \frac{\frac{\pi d^3}{6} \times D \times DF}{D_{DM}} \right) \times 1 + DM \tag{A.12}$$

5’-RACE reads analysis

Genome-wide 5’-rapid amplification of cDNA ends (5’-RACE) reads were first trimmed for quality using Trimmomatic version 0.32 (Bolger *et al*, 2014) and aligned on *M. florum* L1 genome (NC_006055.1) with Bowtie 2 version 2.3.3.1 (Langmead & Salzberg, 2012). A summary of the 5’-RACE library statistics is shown in Appendix Table S1. Reads with a MAPQ below 10 were discarded using samtools version 1.5 (Li *et al*, 2009), and the remaining reads were clipped to retain only a single base at their 5’ extremity, corresponding to putative 5’-end of transcripts. The strand-specific coverage at each genomic position was calculated and normalized according to the number of millions of mapped reads using Bedtools genomecov version 2.27.1 (Quinlan & Hall, 2010), resulting in RSPM values. 5’-RACE peaks with a RSPM signal equal or higher than the average plus one standard deviation single base signal calculated over the entire genome (>=10.92, obtained using 1 kb windows sliding over 100 bp) were considered significant and kept for further analysis (1514 peaks). Significant peaks located at 10 bp or less of each other were merged to retain only the peak with the highest associated RSPM signal, corresponding to a putative transcription start site (TSS). A total of 605 putative TSSs were identified. Promoter motifs were searched by extracting the DNA sequence surrounding each putative TSS (-45 to +5

bp relative coordinates) and submitting it to MEME version 5.0.3 (Bailey & Elkan, 1994) using the zero or one motif per sequence option with a minimum motif length of 40 bp. The presence of promoter motifs nearby significant 5'-RACE peaks was further analyzed using MAST version 5.0.3 (Bailey & Gribskov, 1998) and the identified MEME motif to validate MEME hits and recover putative TSSs potentially lost through the merging procedure. Only MAST hits separated by 3 to 9 bp from a significant peak were kept. This resulted in the addition of eight putative TSSs to the 605 initially identified. To circumvent the misalignment of reads at the chromosome start position, the 5'-RACE reads were realigned on the L1 chromosome sequence linearized at position 397,159 instead of 0, and the whole analysis procedure was repeated. This allowed us to identify an additional TSS located in the intergenic region upstream the *dnaA* gene (peg.1/*mfl001*). This TSS was added to Dataset EV1 and considered for transcription units reconstruction.

Supplementary Text

Genetic context of gTSSs and iTSSs

In total, 432 different motif-associated TSSs were identified by 5'-RACE (see Dataset EV1). 337 of them were located within intergenic regions of the chromosome (gTSSs). Intergenic regions can be divided into three types according to the topology of the neighbouring genes; divergent, convergent, and parallel (Fig. EV3A). Overall, intergenic regions containing gTSSs were significantly larger than those without any gTSS (Fig. EV3B). Most of gTSSs (71.5%) were comprised within parallel intergenic regions as they constitute the most abundant type present in the genome (Fig. EV3C). Conversely, only one case of gTSS was observed in convergent intergenic regions (0.3%), the rest of gTSSs being located within divergent counterparts (28.2%). Nonetheless, divergent intergenic regions most frequently contained gTSSs (96.2%) relative to

their total number of instances in the genome (Fig. EV3D). In contrast, only about half (43.5%) of the parallel intergenic regions contained at least one gTSS. As expected, divergent intergenic regions positive for gTSSs contained most of the time two instances per region, generally disposed back-to-back (Fig. EV3E). Remarkably, these sometimes displayed two overlapping -10 promoter boxes (Fig. EV3F). In comparison, more than 95% of positive parallel regions showed only a single gTSS occurrence (Fig. EV3E).

The remaining motif-associated TSSs (95 out of 432) were positioned within predicted coding regions of the chromosome (iTSSs). In total, 86 out of 720 *M. florum* genes were shown to contain motif-associated iTSSs (Fig. EV3D), with one iTSS per gene in more than 90% of all instances (Fig. EV3E). iTSSs can be separated in two distinct groups based on the orientation of the gene in which they are located: p-iTSSs, same orientation; a-iTSSs, opposite orientation (Fig. EV4A). The majority of motif-associated iTSSs identified in this study consisted of p-iTSSs (71 out of 95), a-iTSSs representing only 5.6% of all TSSs (24 out of 433) (Fig. 3D). iTSSs can be further categorized according to the orientation of the most immediate downstream gene, i.e. whether or not a gene is appropriately oriented to be expressed from a given iTSS (Fig. EV4A). Interestingly, most p-iTSSs were located upstream of genes transcribed on the same strand, contrasting with a-iTSSs predominantly facing their nearest downstream gene (Fig. EV4B). p-iTSSs were also found to be enriched near the end of their overlapping gene, suggesting that they could be involved in the transcription of downstream genes (Fig. EV4C). In fact, several instances of p-iTSSs separated by less than 100 bp from the next correctly oriented downstream gene could be observed (see Fig. EV4D for a visual example). Curiously, a total of nine p-iTSS (out of 71) were also precisely located on the first base of translation start codons, suggesting the transcription of leaderless mRNA (Fig. EV4C). A visual example of such as case is presented in Figure EV4E.

Supplementary Figures

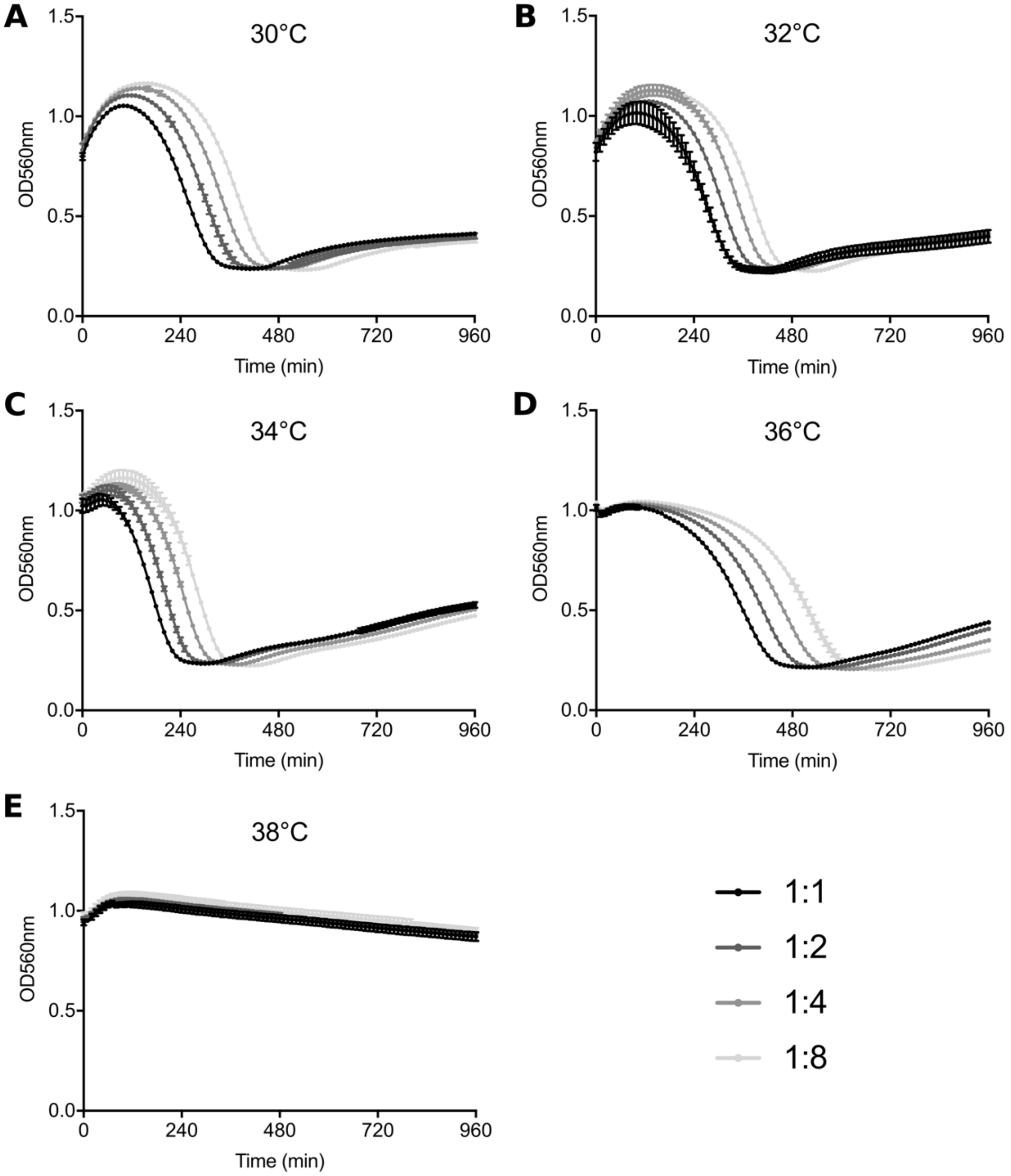

Figure S1. Raw growth curves (OD_{560nm}) of colorimetric assays used to measure the doubling time of *M. florum* incubated at A) 30°C, B) 32°C, C) 34°C, D) 36°C, and E) 38°C. The dots and error bars represent the mean and standard deviation values obtained from three technical replicates.

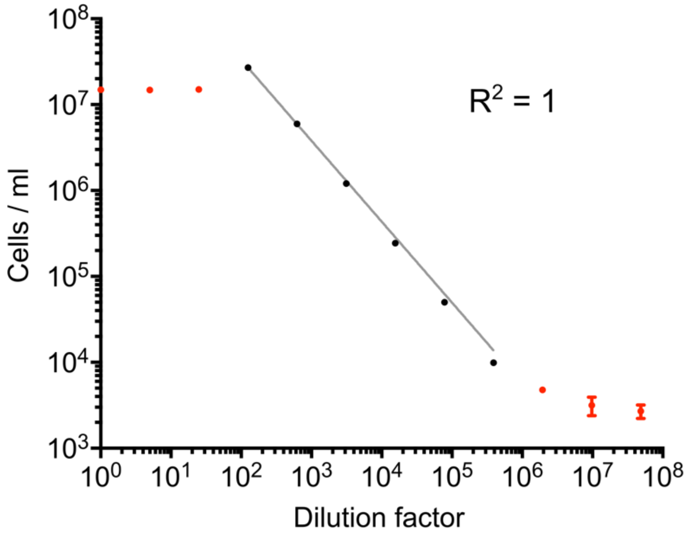

Figure S2. Relationship between *M. florum* cell concentrations measured by flow cytometry (FCM) and culture dilutions performed in PBS1X. A log-log nonlinear regression is shown (gray line), as well as the associated correlation coefficient (R^2). Data points outside the nonlinear regression are colored in red. The Dots and error bars represent the mean and standard deviation values obtained from technical duplicates.

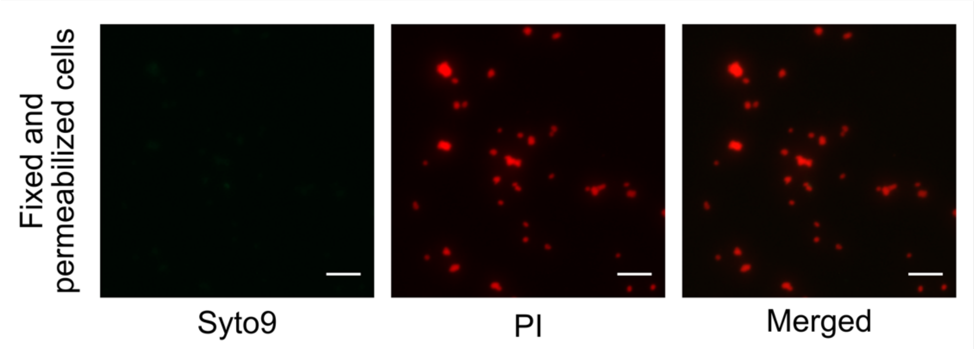

Figure S3. Representative image of fixed and permeabilized *M. florum* cells, double stained with SYTO 9 and propidium iodide (PI), observed by widefield fluorescence microscopy. Scale bar: 5 µm.

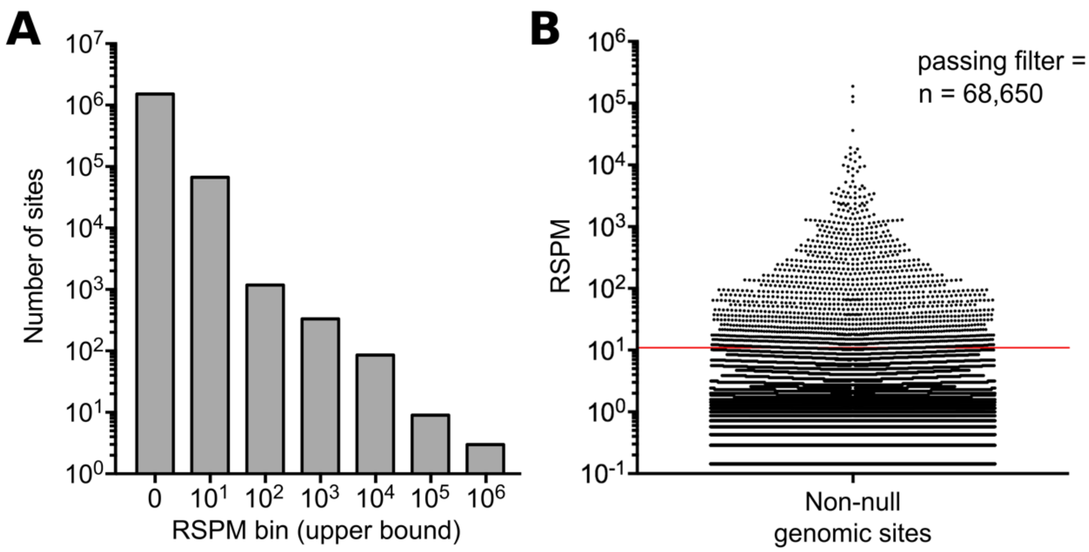

Figure S4. Analysis of 5’-RACE signal intensity. A) Frequency distribution of the 5’-RACE signal intensity observed at each genomic position for both DNA strands. Signal intensity was calculated according to the number of read starts per million of mapped reads (RSPM). RSPM bins are log-scale, and the upper bound value of each bin is shown. B) RSPM signal intensity of all non-null genomic positions (68,650 sites). The threshold value (10.92) used to discriminate significant 5’-RACE peaks from background noise is shown by a red line (see Appendix Material and Methods for further details). A total of 1,514 sites were considered significant.

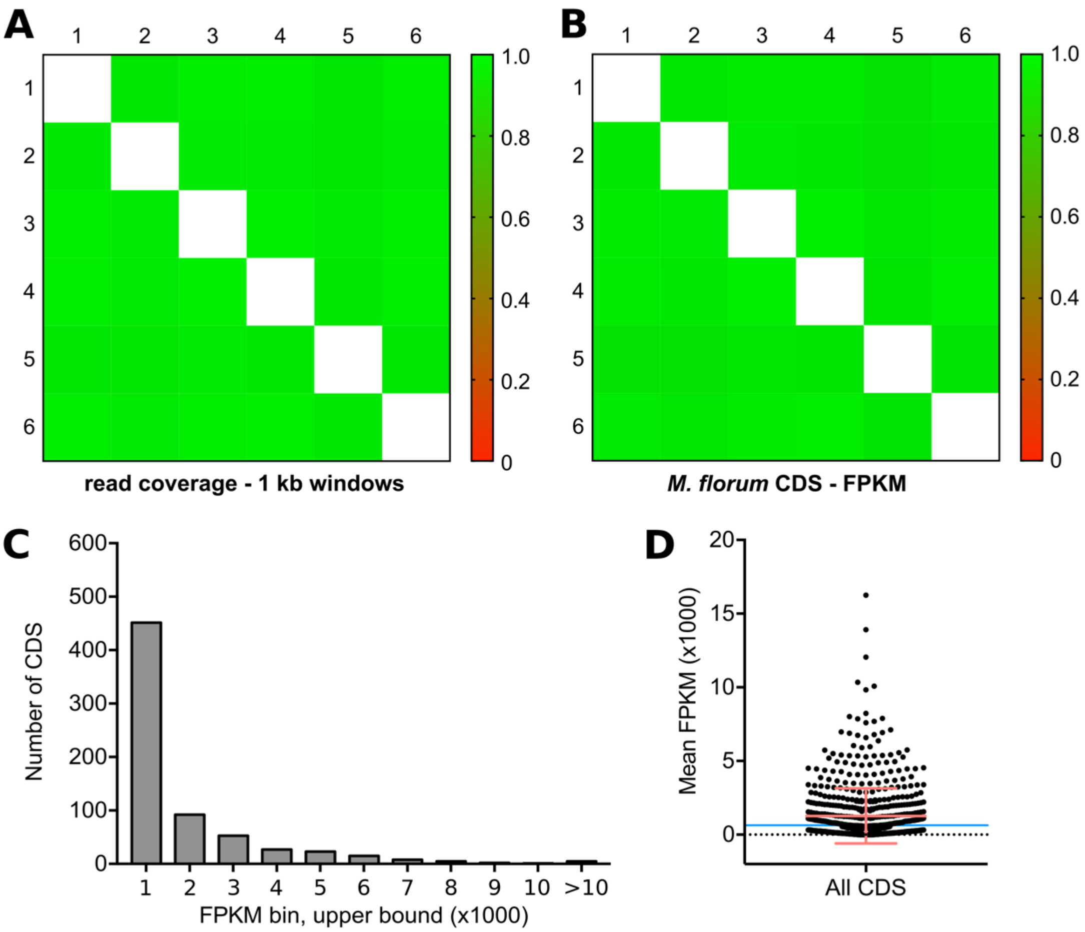

Figure S5. RNA-seq related correlations and distributions. A) Pearson correlation heatmap of RNA-seq read coverage calculated from the different library replicates using non-overlapping 1 kb windows. B) Same as A but using the number of fragments per kilobase per million of mapped reads (FPKM) calculated for *M. florum* protein-coding gene (n=685). C) Frequency distribution of the mean FPKM values of *M. florum* coding sequences (n=685). The upper bound value of each FPKM bin is shown. D) Scatter plot showing the mean FPKM value calculated for each *M. florum* coding sequence. The mean and corresponding SD are shown. The blue line indicates the theoretical FPKM value obtained if all the reads were equally distributed across the genome (FPKM=630).

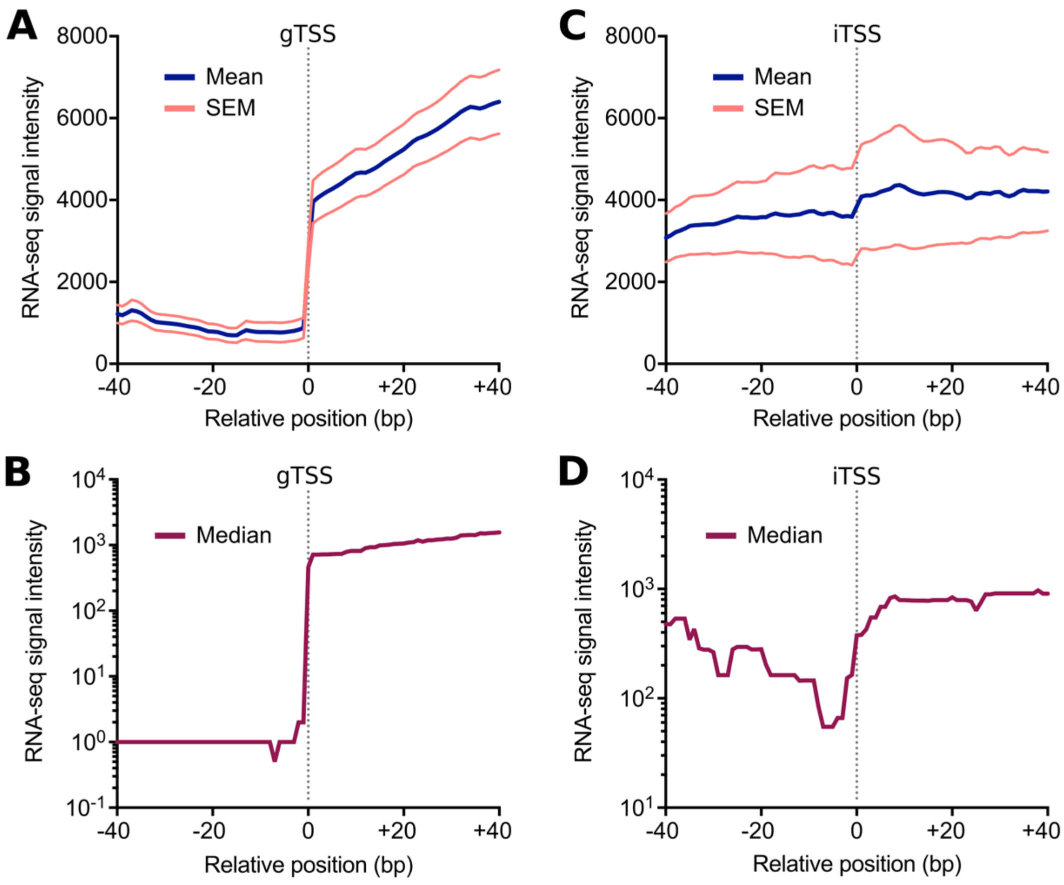

Figure S6. RNA-seq aggregate profiles of TSS types. A) Aggregate profile showing the mean RNA-seq read coverage observed at and around all motif-associated gTSSs identified in this study. The calculated SEM is also shown. The aggregate profile was centered on the gTSSs coordinates (relative position 0 bp), indicated by a gray dashed line. B) Same as A but showing the median value at each position instead of the mean and SEM. C) and D) Identical to A and B, but for motif-associated iTSSs.

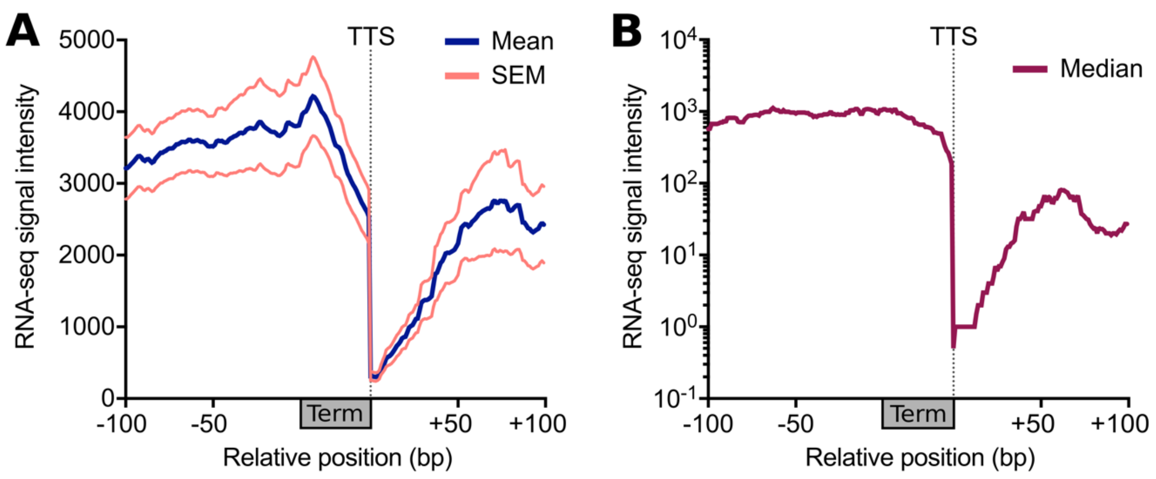

Figure S7. RNA-seq aggregate profiles of Rho-independent terminators predicted in this study. A) Aggregate profile showing the mean RNA-seq read coverage observed for all predicted terminators and their surrounding DNA regions. The calculated SEM is also shown. The aggregate profile was centered on the terminators start and stop coordinates. The predicted transcription termination site (TTS) is indicated by a gray dashed line. B) Same as A but showing the median value at each position instead of the mean and SEM.

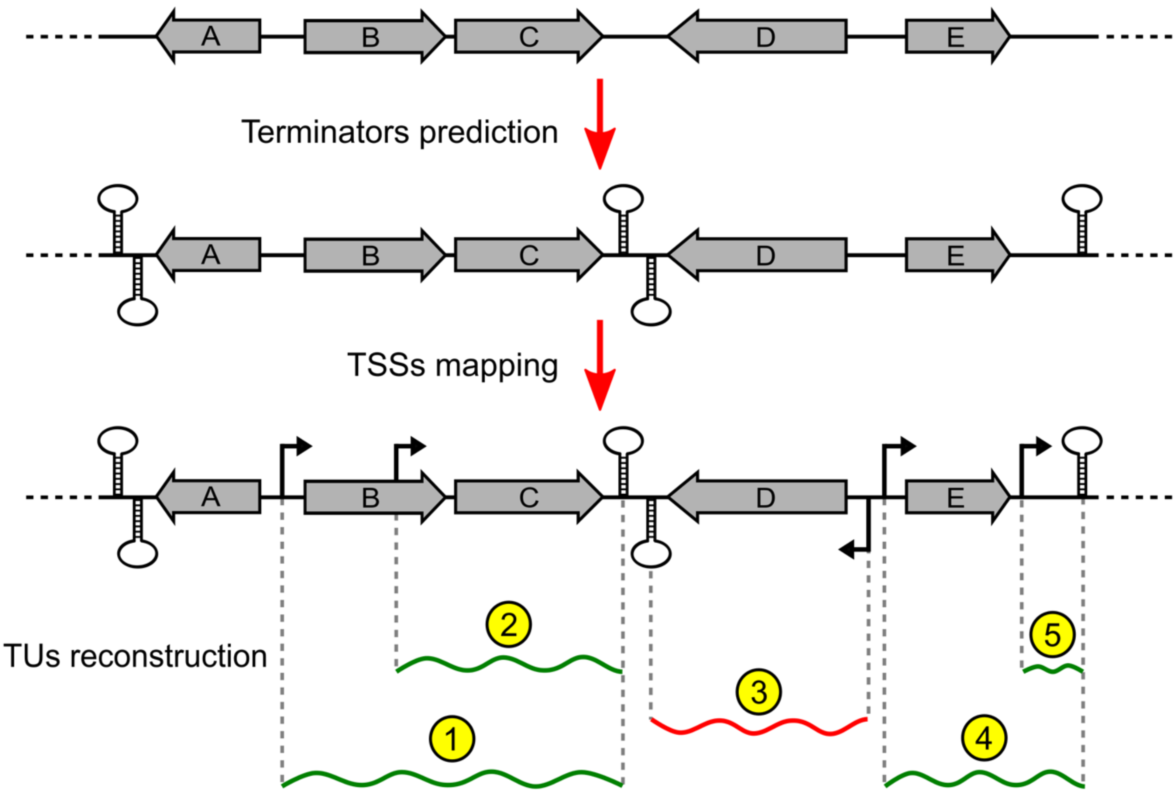

Figure S8. Summary of transcription unit reconstruction procedure. First, Rho-independent terminators were predicted from the DNA sequence and genes annotation as described previously (de Hoon *et al*, 2005), creating strand-specific term-to-term scaffolds. Motif-associated TSSs were then mapped onto the scaffolds, and all possible transcription units (TUs) were reconstructed. Depending on the context, some TUs may contain a single gene (TUs 2, 3, and 4), many genes (TU 1), or no gene at all (TU 5; non-coding TU). Certain TUs may also partially overlap other genes if they originate from iTSSs (TU 2). Genes not included in at least one TU and therefore not associated with any TSS were classified as orphan genes (gene A).

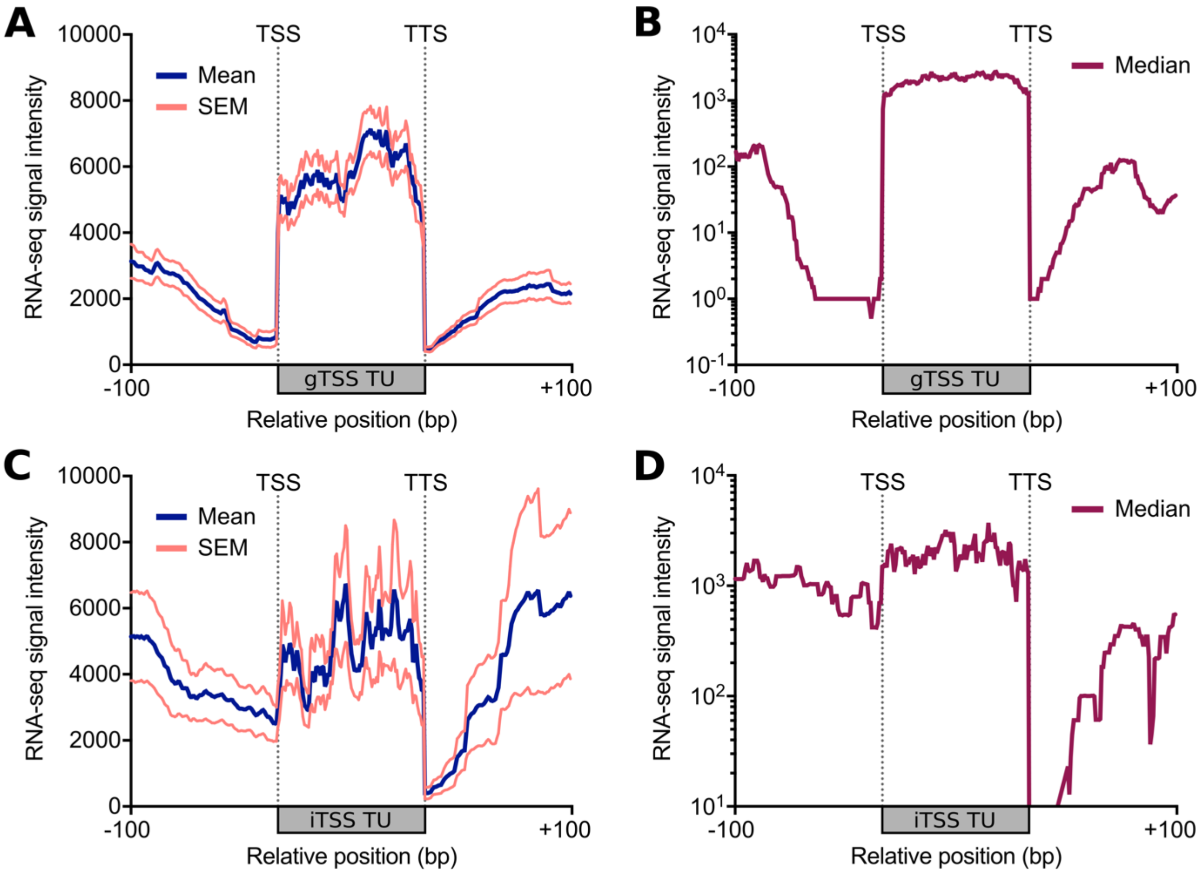

Figure S9. RNA-seq aggregate profiles of gTSS and iTSS transcription units (TUs). A) Aggregate profile showing the mean RNA-seq read coverage observed for all gTSS TUs and their surrounding DNA regions. The calculated SEM is also shown. The aggregate profile was centered on the TUs start and stop coordinates, corresponding to transcription start site (TSS) and termination site (TTS), respectively. B) Same as A but showing the median value at each position instead of the mean and SEM. C) and D) Identical to A and B, but for iTSS TUs.

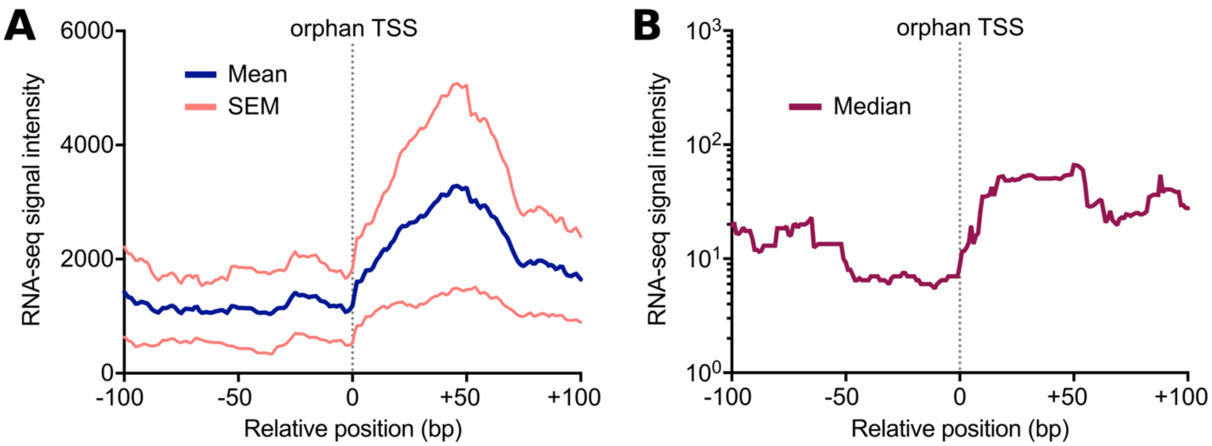

Figure S10. RNA-seq aggregate profiles of orphan TSSs and gTSSs located immediately upstream a predicted terminator. A) Aggregate profile showing the mean RNA-seq read coverage and the associated SEM values. The aggregate profile was centered on the TSSs coordinates (relative position 0 bp), indicated by a gray dashed line. B) Same as A but showing the median value at each position instead of the mean and SEM.

Supplementary Tables

Table S1. Statistical summary of Illumina sequencing libraries prepared in this study.

Library type	Sequencing type	Replicate	Total reads (single)	Reads passing quality filters	Aligned reads (MAPQ>=10)	Genome coverage
5’-RACE	SE 40 bp	1	10,234,272	9,442,841 (92%)	6,961,595 (74%)	~350X
RNAseq	PE 50 bp	1	16,089,680	14,003,252 (87%)	13,049,819 (93%)	~820X
		2	16,531,090	14,234,385 (86%)	12,649,001 (89%)	~800X
		3	16,788,638	14,493,548 (86%)	13,605,303 (94%)	~860X
		4	17,389,570	15,067,903 (87%)	14,377,039 (95%)	~910X
		5	18,566,270	15,929,927 (86%)	14,980,959 (94%)	~940X
		6	15,247,438	13,105,485 (86%)	12,160,110 (93%)	~770X

Supplementary References

Bailey TL & Elkan C (1994) Fitting a mixture model by expectation maximization to discover motifs in biopolymers. *Proc Int Conf Intell Syst Mol Biol* 2: 28–36

Bailey TL & Gribskov M (1998) Combining evidence using p-values: application to sequence homology searches. *Bioinformatics* 14: 48–54

Bolger AM, Lohse M & Usadel B (2014) Trimmomatic: A flexible trimmer for Illumina sequence data. *Bioinformatics* 30: 2114–2120

Fahy E, Subramaniam S, Murphy RC, Nishijima M, Raetz CRH, Shimizu T, Spener F, van Meer G, Wakelam MJO & Dennis EA (2009) Update of the LIPID MAPS comprehensive classification system for lipids. *J Lipid Res* 50: S9–S14

de Hoon MJL, Makita Y, Nakai K & Miyano S (2005) Prediction of transcriptional terminators in *Bacillus subtilis* and related species. *PLoS Comput Biol* 1: e25

Langmead B & Salzberg SL (2012) Fast gapped-read alignment with Bowtie 2. *Nat Methods* 9: 357–9

Li H, Handsaker B, Wysoker A, Fennell T, Ruan J, Homer N, Marth G, Abecasis G & Durbin R (2009) The Sequence Alignment/Map format and SAMtools. *Bioinformatics* 25: 2078–2079

Quinlan AR & Hall IM (2010) BEDTools: a flexible suite of utilities for comparing genomic features. *Bioinformatics* 26: 841–842
