# Supplementary material for: Integrative characterization of the near‐minimal bacterium Mesoplasma florum
Source: Mol Syst Biol. 2020 Dec 17;16(12):e9844. doi: 10.15252/msb.20209844 (PMC7745072; doi:10.15252/msb.20209844)
Supplement: Supplementary file 3 — Expanded View Figures PDF [file MSB-16-e9844-s002.pdf]

Expanded View Figures

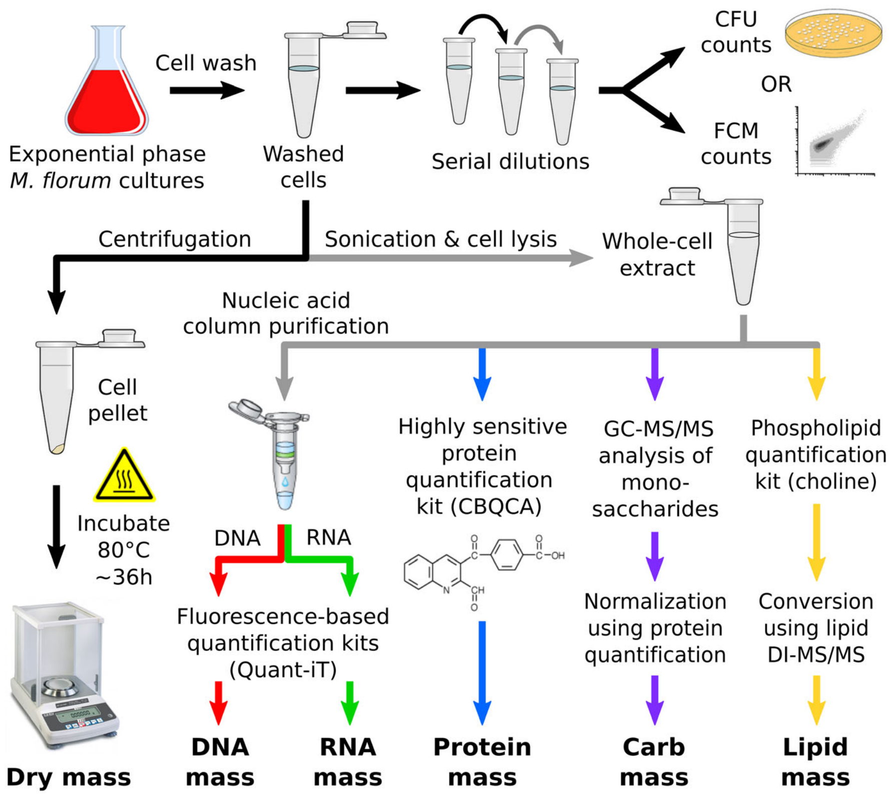

Figure EV1. Overview of the experimental procedures used to determine the total dry mass of *M. florum* as well as the mass of its principal cellular macromolecules.

Each constituent is quantified using high sensitivity assays or mass spectrometry methods. Quantification results are normalized according to the number of cells used for each experiment. See Appendix Supplementary Methods for further details.

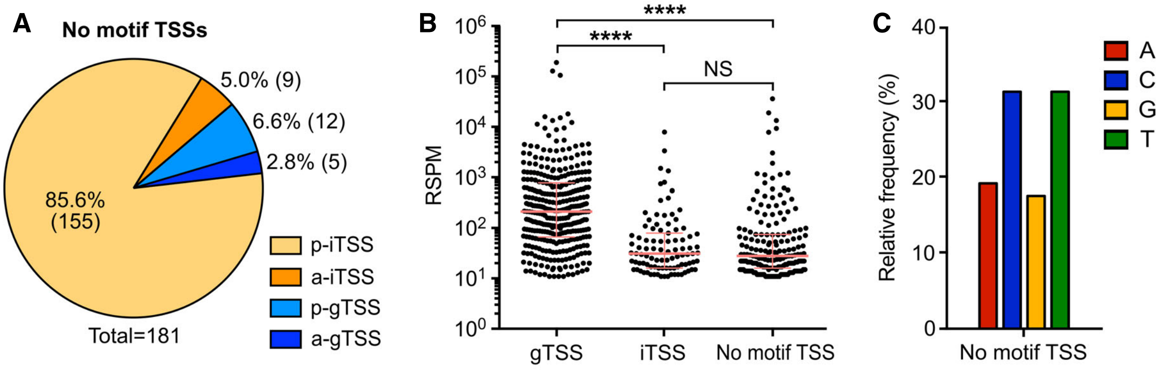

Figure EV2. Principal characteristics of transcription start sites (TSSs) not associated with the *M. florum* promoter motif.

A Localization and orientation of TSSs without a MEME or MAST promoter motif. p-gTSS, parallel intergenic TSS; a-gTSS, antiparallel intergenic TSS; p-iTSS, parallel internal TSS; a-iTSS, antiparallel internal TSS. For gTSSs, the orientation was defined according to the closest downstream gene, while the overlapping gene was used in the case of iTSSs.

B Comparison of the read start per million of mapped reads (RSPM) signal intensity of gTSSs, iTSSs, and TSSs without any promoter motif. The median and interquartile range are shown for each group. Distributions were compared using a Kruskal–Wallis test with Dunn's multiple comparison post-test (****P-value < 0.0001).

C Nucleotide identity at the transcription initiation site (+1) for TSSs without a promoter motif.

© 2020 The Authors

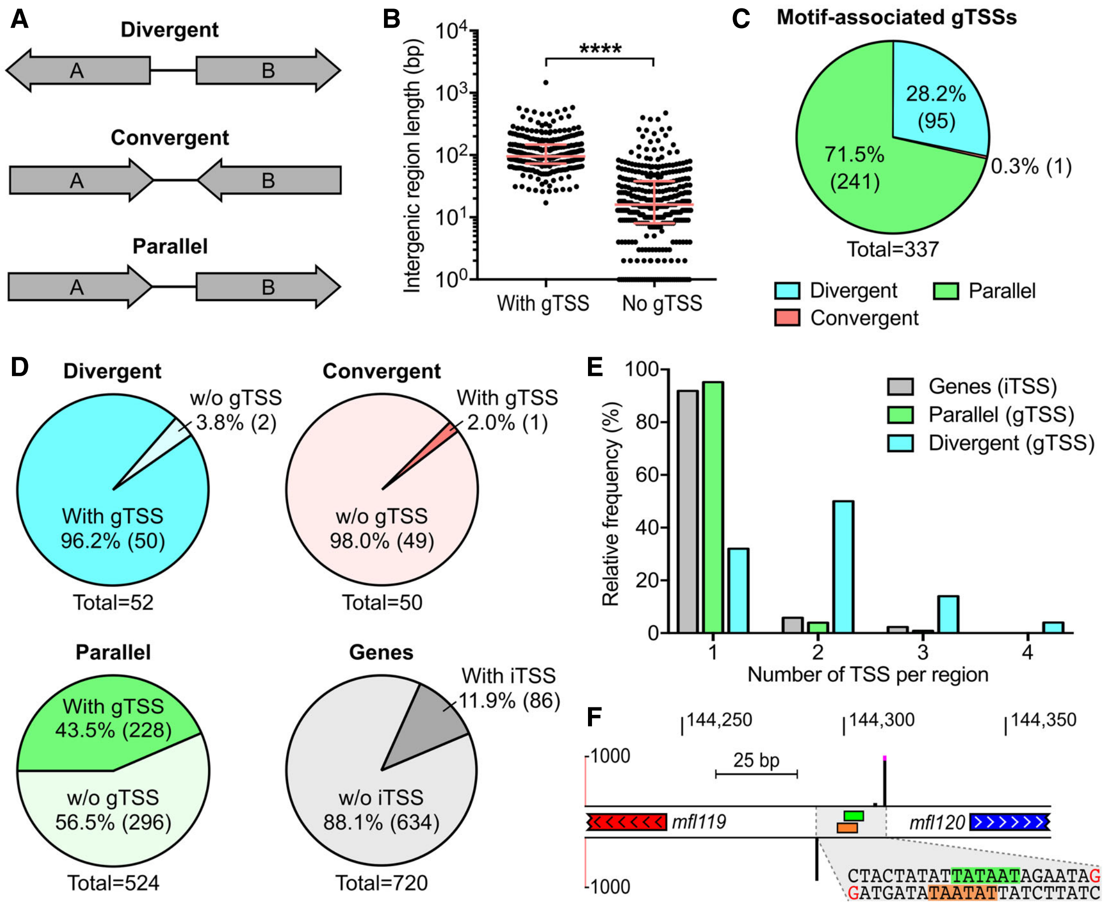

Figure EV3. Additional information concerning the genetic context of motif-associated TSSs.

A Types of intergenic regions based on surrounding genes orientation.

B Length of intergenic regions with or without gTSS. The median and interquartile range are shown for each group. Distributions were compared using a Mann–Whitney test (two-sided, ****P-value < 0.0001).

C Total number of gTSSs for each of the three intergenic region groups depicted in A.

D Proportion of divergent, convergent, and parallel intergenic regions hit by at least one gTSS relative to their respective total number across the genome. The proportion of genes hit by iTSSs is also shown.

E Relative frequency distribution of the number of motif-associated TSSs detected per gene, parallel intergenic region or divergent intergenic region.

F Genomic locus showing a representative case of two divergent genes expressed from two back-to-back overlapping promoters identified by 5′-RACE. Genomic coordinates are indicated at the top of the panel. Strand-specific 5′-RACE signals are shown by black bars (0–1,000 read starts scale). Peaks above 1,000 read starts are cut and marked by fuchsia dots. The position of −10 boxes attributed to 5′-RACE peaks are indicated by green and orange rectangles for positive and negative DNA strands, respectively. The genomic coordinates containing the identified TSSs and −10 boxes is enlarged and its corresponding DNA sequence is illustrated. Bases corresponding to +1 sites are colored in red. Bases corresponding to the −10 boxes are highlighted in green and orange for positive and negative DNA strands.

© 2020 The Authors

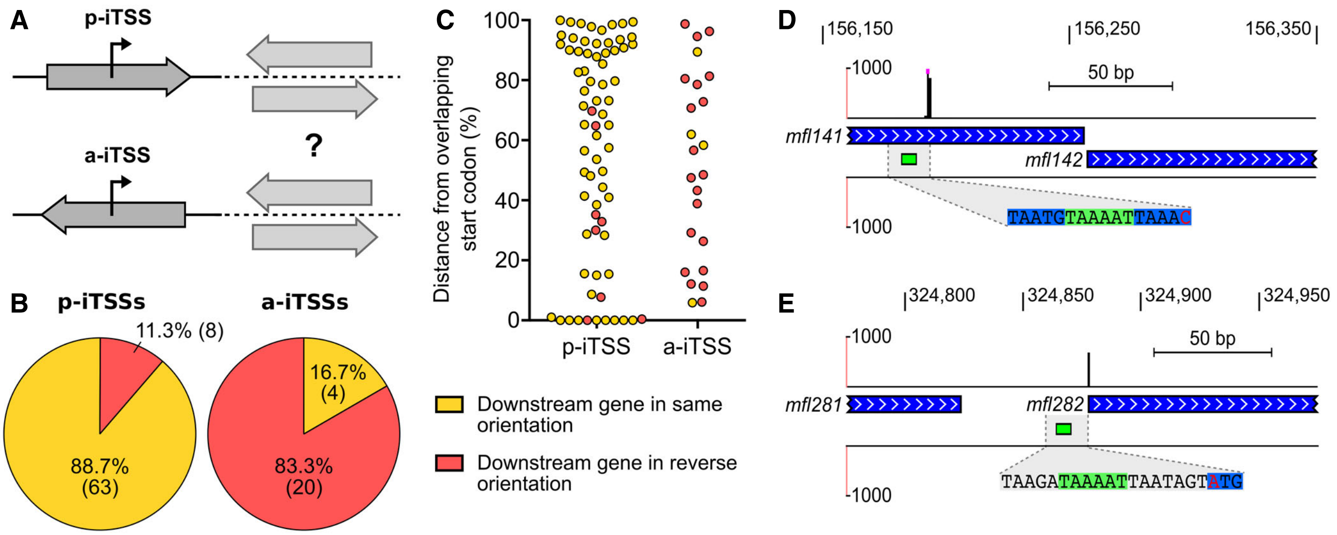

Figure EV4. Additional information about the genetic context of motif-associated iTSSs.

A Classification of iTSSs according to the orientation of the overlapping gene in which they are located. p-iTSS, parallel internal TSS; a-iTSS, antiparallel internal TSS. Depending on the orientation of downstream genes, both TSS types could contribute to their expression.

B p-iTSSs and a-iTSSs orientation relative to the nearest downstream gene.

C Distance from overlapping gene start codon for p-iTSSs and a-iTSSs. Distance was normalized according to the overlapping gene length. Yellow and red dots indicate iTSSs located upstream genes of the same and reverse orientation, respectively.

D, E Genomic loci showing representative cases of p-iTSS located at less than 100 bp from the most immediate downstream gene (D) and p-iTSS located directly on a translation start codon (E). Details are as in Fig EV3F.

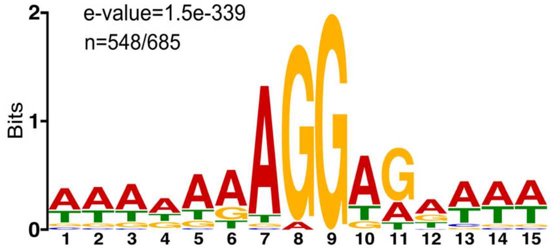

Figure EV5. *Mesoplasma florum* ribosome binding site motif.

The motif was determined from the DNA region located immediately upstream (≤ 20 bp) the translation initiation codon of every reference open reading frame. A total of 548 upstream regions were included in the motif (out of 685).

© 2020 The Authors
