# Supplementary material for: Integrative characterization of the near‐minimal bacterium Mesoplasma florum
Source: Mol Syst Biol. 2020 Dec 17;16(12):e9844. doi: 10.15252/msb.20209844 (PMC7745072; doi:10.15252/msb.20209844)
Supplement: Supplementary file 4 — Table EV1 [file MSB-16-e9844-s003.docx]

**Table EV1.** Genome features and intracellular levels of important molecules and complexes in *M. florum* L1 compared to other selected species.

|  | ***M. florum* L1** | ***M. mycoides capri* (JCVI-syn3A)** | ***M. pneumoniae* M129** | ***E. coli*** |
| --- | --- | --- | --- | --- |
| **GenBank Accession** | AE017263.1 | CP016816.2 | U00089.2 | U00096.3 (MG1655) |
| Genome size (bp) | 793,224 | 543,379 | 816,394 | 4,641,652 |
| Genes | 720^(a)^ | 493^(b)^ | 733^(c)^ | 4538^(g)^ |
| Coding sequences | 685^(a)^ | 452^(b)^ | 689^(c)^ | 4336^(g)^ |
| RNA genes | 35^(a)^ | 38^(b)^ | 44^(c)^ | 202^(g)^ |
| **Molecules per cell** |  |  |  |  |
| Chromosome | 2.1 | 1^(b)^ | NA | 2^(h)^  2.3^(i)^ |
| Total RNA | 23,320 | NA | 4,430 | 261,400^(h)^  258,000^(i)^ |
| rRNA | 4,900 | NA | 900^(d)^ | 60,000^(h)^  54,000^(i)^ |
| tRNA | 18,000 | NA | 3,300^(d)^ | 200,000^(h,i)^ |
| mRNA | 420 | NA | 230^(e)^ | 1,400^(h)^  4,000^(i)^ |
| Protein | 250,000 | 77,000^(b)^ | 130,000^(d)^ | 3,000,000^(h)^  3,600,000^(i)^  3,000,000-4,000,000^(j)^ |
| Ribosome | 1,600-2,100 | 340-670^(b)^ | 300^(d)^  190^(c)^  140^(f)^ | 6,800-72,000^(h)^  18,000^(i)^  30,000-70,000^(k)^ |
| Core RNA polymerase | 270 | 380^(b)^ | 300^(f)^ | 1,500-11,400^(h)^  2,000-10,000^(k)^  2,600-13,000^(l)^ |
| σ^70^ | 230 | 230^(b)^ | NA | 4,700-17,000^(l)^ |
| **Average cell volume (µm^3^)** | 0.090 | 0.034^(b)^ | 0.067^(d)^ | 1.0^(h,i)^  1.3-3.0^(k)^ |
| **Molecules per µm^3^** | | | | |
| Chromosome | 23.3 | 29.4^(b)^ | NA | 2^(h)^  2.3^(i)^ |
| Total RNA | 260,000 | NA | 65,400 | 261,400^(h)^  258,000^(i)^ |
| rRNA | 54,000 | NA | 13,000^(d)^ | 60,000^(h)^  54,000^(i)^ |
| tRNA | 200,000 | NA | 49,000^(d)^ | 200,000^(h,i)^ |
| mRNA | 4,700 | NA | 3,400^(e)^ | 1,400^(h)^  4,000^(i)^ |
| Protein | 2,800,000 | 2,260,000^(b)^ | 1,900,000^(d)^ | 3,000,000^(h)^  3,600,000^(i)^  3,000,000-4,000,000^(j)^ |
| Ribosome | 18,000-24,000 | 10,000-20,000^(b)^ | 4,500^(d)^  2,800^(c)^  2,100^(f)^ | 18,000^(i)^  25,000-31,000^(k)^ |
| Core RNA polymerase | 3,000 | 11,000^(b)^ | 4,500^(f)^ | 1,500-11,400^(h)^  1,800-3,500^(k)^  2,600-13,000^(l)^ |
| σ^70^ | 2,600 | 6,800^(b)^ | NA | 4,700-17,000^(l)^ |

### ^(a)^RAST genome annotation, Baby *et al.* 2018.

### ^(b)^Breuer *et al.* 2019.

### ^(c)^Wodke *et al.* 2013.

### ^(d)^Yus *et al.* 2009.

### ^(e)^Weiner *et al.* 2003.

### ^(f)^Kuhner *et al.* 2009.

### ^(g)^Keseler et al. 2017.

### ^(h)^*E. coli* strain B/r, Bionumbers 2015.

### ^(i)^CCDB database, unspecified strain.

### ^(j)^Milo 2013.

### ^(k)^Bakshi *et al.* 2012.

### ^(l)^Grigorova *et al.* 2006.
